# Overexpression of E2F1 in human gastric carcinoma is involved in anti-cancer drug resistance

**DOI:** 10.1186/1471-2407-14-904

**Published:** 2014-12-03

**Authors:** Lin-Hai Yan, Wei-Yuan Wei, Wen-Long Cao, Xiao-Shi Zhang, Yu-Bo Xie, Qiang Xiao

**Affiliations:** Department of Gastrointestinal Surgery, Affiliated Tumor Hospital of Guangxi Medical University, Nanning, Guangxi Zhuang Autonomous Region, 530021 China; Department of Surgery, The First Affiliated Hospital of Guangxi Medical University, Nanning, Guangxi Zhuang Autonomous Region, 530021 China; Department of Anesthesiology, The First Affiliated Hospital of Guangxi Medical University, Nanning, Guangxi Zhuang Autonomous Region, 530021 China

**Keywords:** E2F1 transcription factor, Lentiviral vector, Gastric carcinoma, Drug resistance, Murine model

## Abstract

**Background:**

Routine chemotherapy often cannot achieve good therapeutic effects because of multidrug resistance (MDR). MDR is frequently caused by the elevated expression of the MDR1 gene encoding P-glycoprotein (P-gp). E2F1 is a frequently overexpressed protein in human tumor cells that increases the activity of the MDR1 promoter, resulting in higher P-gp levels. The upregulation of P-gp might contribute to the survival of tumor cells during chemotherapy. E2F1 confers anticancer drug resistance; however, we speculate whether E2F1 affects MDR through other pathways. This study investigated the possible involvement of E2F1 in anticancer drug resistance of gastric carcinoma *in vitro* and *in vivo*.

**Methods:**

A cisplatin-resistant SGC7901/DDP gastric cancer cell line with stable overexpression of E2F1 was established. Protein expression levels of E2F1, MDR1, MRP, TAp73, GAX, ZEB1, and ZEB2 were detected by western blotting. The influence of overexpression of E2F1 on anticancer drug resistance was assessed by measuring IC50 of SGC7901/DDP cells to cisplatin, doxorubicin, and 5-fluorouracil, as well as the rate of doxorubicin efflux, apoptosis, and cell cycle progression detected by flow cytometry. We determined the *in vivo* effects of E2F1-overexpression on tumor size in nude mice, and apoptotic cells in tumor tissues were detected by deoxynucleotidyl transferase-mediated dUTP-biotin nick end labeling and hematoxylin and eosin staining.

**Results:**

The SGC7901/DDP gastric cancer cell line stably overexpressing E2F1 exhibited significantly inhibited sensitivity to cisplatin, doxorubicin, and 5-fluorouracil. Flow cytometry confirmed that the percentage of apoptotic cells decreased after E2F1 upregulation, and that upregulation of E2F1 potentiated S phase arrest of the cell cycle. Furthermore, upregulation of E2F1 significantly decreased intracellular accumulation of doxorubicin. Western blot revealed that E2F1 upregulation suppressed expression of GAX, and increased the expression of MDR1, MRP, ZEB1, TAp73, and ZEB2.

**Conclusions:**

Overexpression of E2F1 promotes the development of MDR in gastric carcinoma, suggesting that E2F1 may represent an efficacious target for gastric cancer therapy.

## Background

Resistance to anti-neoplastic agents is the major cause of therapy failure, leading to disease recurrence and metastasis. The molecular genetic basis of resistance to cancer therapeutics is generally complex, involving multiple processes such as drug transport, drug metabolism, DNA repair and apoptosis [1]. Emerging evidence suggests that the mechanisms of multidrug resistance (MDR) are closely associated with the overexpression of P-gp encoded by the MDR1 gene. In tumor cells, P-gp acts as a drug efflux pump that actively transports drugs from the inside to the outside of cancer cells and thus prevents the intracellular accumulation of anticancer drugs necessary for cytotoxic activity [2]. However, the factors that regulate the chemoresistance of gastric carcinoma remain poorly understood.

E2F1 is a unique member of the E2F family of proteins as it is involved in cell cycle progression and apoptosis induction in response to DNA damage through its capacity to activate p53/p73 death pathways [3, 4]. A previous study reported that deregulated E2F1 acts as a driving force in melanoma progression and promotes tumor invasion and metastasis independently from its other cellular activities. This aggressive behavior of the transcription factor in malignant cells is partially mediated through the induction of the epidermal growth factor receptor pathway [5]. Most importantly, E2F1 plays a critical role in the malignant phenotypes of some cancers. Previous studies reported that E2F1 could affect cell proliferation and apoptosis and that E2F1 may be involved in regulating MDR in some cancers [6, 7]. In addition, E2F1 stimulates the promoter of the MDR1 gene, resulting in increased expression and higher levels of P-gp, thus possibly contributing to the development of MDR [8]. Furthermore, its downregulation suppresses MDR in gastric carcinoma cells *in vitro* and *in vivo* [9]. Although this evidence implies that E2F1 is associated with carcinogenesis and development of MDR, the precise role of E2F1 in MDR of gastric carcinoma remains largely unexplored.

To define the role of E2F1 in multidrug-resistant gastric carcinoma, we generated gastric carcinoma cells that stably express E2F1 and evaluated changes in IC50, the rate of doxorubicin efflux, cell cycle, and apoptosis. We also examined the expression of genes associated with apoptosis and multidrug resistance, including GAX, TAp73, MDR1, MRP, ZEB1, and ZEB2. Moreover, we investigated the effects of E2F1 upregulation on the growth and apoptosis of SGC7901/DDP cells *in vivo*.

## Methods

### Reagents and drugs

Adriamycin (ADR) (KEYGEN Biotech, China) was diluted in phosphate-buffered saline (PBS) (2 mg/ml). Cis-diamminedichloroplatinum (cisplatin, DDP) (Qilu Pharmo Co. Ltd, China) was resuspended in PBS (1 mg/ml) and stored at −20°C. 5-fluorouracil (5-FU) (KEYGEN Biotech) was added in solution (25 mg/ml) and stored at room temperature. E2F1, GAX, TAp73, MDR1, MRP, ZEB1, ZEB2 and glyceraldehyde 3-phosphate dehydrogenase (GAPDH) antibodies were obtained from Santa Cruz Biotechnology (Santa Cruz, CA, USA). All other chemicals were of the highest available commercial grade.

### Cell culture

Cisplatin-resistant SGC7901 (SGC7901/DDP) cells were purchased from KEYGEN Biotech. SGC7901/DDP cells were cultured in RPMI-1640 (Hyclone) supplemented with 10% fetal bovine serum (FBS) (Hangzhou Sijiqing Biotech, Co. Ltd, China) and antibiotics (100 U/ml penicillin and 100 mg/ml streptomycin) in a humidified 5% CO_2_ atmosphere at 37.8°C (Thermo). Cisplatin (0.6 μg/mL) was supplemented in the medium for SGC7901/DDP cell culture to maintain the drug-resistance phenotype.

### Establishment of stable cell lines

The PLNCX lentiviral vector (LV-GFP) purchased from Shanghai Cancer Institute, China was used to construct the E2F1 overexpression vector. The construction of LV-E2F1-GFP and transfection of SGC7901/DDP gastric carcinoma cells with LV-E2F1-GFP or LV-GFP have been previously described [9]. SGC7901/DDP cells were seeded in six-well plates with antibiotic-free medium. The cells were divided into three groups: E2F1 group (SGC7901/DDP + E2F1), GFP group (SGC7901/DDP + GFP), and NC (negative control) group (SGC7901/DDP). After 24 h incubation, cells were infected with the indicated viral supernatant at a multiplicity of infection of 120 PFU per cell (MOI = 120), and stably transfected cell lines were obtained by culturing transfected cells in the presence of 700 mg/mL G418 (Invitrogen, Carlsbad, CA, USA) for 2–3 weeks.

### Semiquantitative reverse-transcriptase polymerase chain reaction

Total RNA was isolated using the AxyPrep™ Purification Kit (Axygen, USA) according to the manufacturer’s instructions. The total RNA concentration and quality were measured with a Nanodrop 2000 micro-volume spectrophotometer (Thermo Scientific, USA) by absorbance measurements. RNA integrity was analyzed by 2% agarose gel electrophoresis and ethidium bromide staining. First-strand cDNA was synthesized from 3000 ng of total RNA using the RevertAidHMinus First Strand cDNA synthesis kit (Fermentas, USA) as instructed by the manufacturer. Real-time PCR (RT-PCR) reactions were carried out on an Mx3000P real-time PCR system (Stratagene USA). To create the RT-PCR standard, glyceraldehyde 3-phosphate dehydrogenase (GAPDH) was used as the internal control. The PCR primer sequences were as follows: E2F1 primer sense 5′-CCC AAC TCC CTC TAC CCT-3′ and antisense 5′-CTC CCA TCT CAT ATC CAT CCT G-3′; and GAPDH primer sense 5′-ACC ACA GTC CAT GCC ATC AC-3′ and antisense 5′-TCA CCA CCC TGT TGC TGT A-3′. The PCR products were checked by agarose gel electrophoresis, and the abundance of each mRNA was detected and normalized to that of GAPDH mRNA.

### Western blot, immunoprecipitation and pull-down assays

Cell lysates were prepared in a buffer containing 100 mmol/L NaCl, 10 mmol/L Tris–HCl (pH 7.6), 1 mmol/L EDTA (pH 8.0), 1 μg/mL aprotinin, 100 μg/mL phenylmethylsulfonyl fluoride, and 1% (v/v) NP-40. After protein quantitation using the Lowery protein assay, equal amounts of proteins were separated by SDS-PAGE and blotted onto nitrocellulose membranes by the semi-dry blotting method using a three-buffer system. The membranes were incubated with a dilution of primary antibodies (anti-E2F1: 1:1500, anti-GAX: 1:3000, anti-TAp73: 1:2000, anti-MDR1: 1:3000, anti-MRP: 1:1500, anti-ZEB1: 1:1000, anti-ZEB2: 1:2000), overnight at 4°C. The membrane was washed with TBST and incubated with a peroxidase-conjugated secondary antibody (1:1000) (Santa Cruz Biotechnology) for 1 h. Specific antibody binding was detected using a chemiluminescence detection system (Pierce, Rockford, IL, United States), according to the manufacturer’s recommendations. Western blot film was scanned, and the net intensities of the bands were quantified using Image-QuanT software (Molecular Dynamics, Sunnyvale, CA, United States). After development, the membrane was stripped and reprobed with antibodies against GAPDH (1:1000) or β-actin (1:1500) to confirm equal sample loading. Immunoprecipitation and GST pull-down assays were performed as described previously [10].

### Cytotoxicity assay

Cytotoxicity was determined by Cell Counting Kit-8 (CCK-8) assay (KEYGEN Biotech, China). Cells were seeded in 96-well plates in 100 μl RPMI-1640 medium supplemented with 10% FBS at 5 × 10^4^ cells/well. Cisplatin (0.6 μg/mL) was added in normal growth medium supplemented with FBS. After 48 h incubation, 10 μl CCK-8 reagent was added and culture was continued for 1 h in a humidified atmosphere containing 5% CO_2_. Absorbances at 450 nm were measured by a Microplate Reader (Biotech Company). The relative drug resistance was analyzed compared with IC50 values.

### Measurement of pump rate of doxorubicin by flow cytometry

Cells were inoculated into six-well plates containing 4 mg/mL doxorubicin and cultured at 37°C for 30 min. Flow cytometry was used to measure the fluorescent intensity of doxorubicin in cells with an excitation wavelength of 488 nm and emission wavelength of 575 nm. The cells were then washed twice with fresh culture medium and incubated with the new medium at 37°C for 1 h to detect the retained doxorubicin. Subtraction of the fluorescence retained from the total fluorescence was the fluorescent index of doxorubicin. The procedure was repeated three times and an average value was obtained to calculate the pump rate of doxorubicin. The pump rate of the drug from the cells = (accumulated quantity of doxorubicin – retained quantity of doxorubicin)/accumulated quantity of doxorubicin.

### Apoptosis analysis by flow cytometry

SGC7901/DDP cells (1 × 10^6^) were washed twice with ice-cold PBS, treated with trypsin, and fixed in cold 70% ethanol at 4°C for 30 min. The cell pellet was incubated in a solution containing 10 μl/mL Annexin V-FITC and 10 μl/mL 7-amino-actinomycin D (7-AAD). The cells were analyzed by flow cytometry using an EPICS XL-MCL FACScan (Becton-Dickinson, Mountain View, CA, USA). The data were analyzed with MultiCycle Software for Windows (Phoenix Flow Systems, San Diego, CA, USA).

### Cell cycle analysis by flow cytometry

SGC7901/DDP cells (1 × 10^6^) were washed twice with ice-cold PBS, treated with trypsin, and fixed in cold 70% ethanol at 4°C for 30 min. The cell pellet was incubated in a solution containing 50 ng/mL propidium iodide, 0.2 mg/mL RNase, and 0.1% Triton X-100 at room temperature for 30 min. The cells were analyzed by flow cytometry as described above.

### Effect of LV-E2F1-GFP on promoting MDR of human gastric carcinoma *in vivo*

BALB/c 5-week-old male nude mice (Guangxi Animal Center, Nanning, China) were kept under specific pathogen-free conditions and tended to in accordance with institutional guidelines. All experimental studies were approved by the Guangxi Medical University Animal Care and Use Committee. Approximately 2 × 10^6^ SGC7901/DDP cells were resuspended in 100 μL PBS, and implanted subcutaneously into the flanks of the BALB/c nude mice. The resulting tumor was named the SGC7901/DDP tumor. After 7 days, when the SGC7901/DDP tumor measured 3–5 mm in diameter, the mice were randomly divided into three groups (six mice/per group): E2F1, GFP, and SGC7901/DDP. The animals were administered an intratumoral injection of LV-E2F1-GFP or LV-GFP at a titer of 5 × 10^6^ TU in 100 μL PBS. Injection of an equal volume of PBS was used as a negative control (NC). After the first injection, the animals were re-injected every 2 days. DDP was administered by intraperitoneal injection at a dose of 25 mg/kg, followed by re-administration every 2 days. The tumors were monitored every day and measured every 2 days with a caliper, and the diameters were recorded. The tumor volume (TV) was calculated by the formula: *TV* = *W*^2^ × *L*/2, where *L* is the length and *W* is the width of the tumor. The relative tumor volume (RTV) was calculated by the formula: *RTV* = *V*_t_/*V*_0_ (*V*_0_ is the *TV* at the day when the chemicals were given, and *V*_t_ is the TV of subsequent measurement). The animals were sacrificed 34 days after tumor injection and the tumors were analyzed.

### Hematoxylin and eosin staining and deoxynucleotidyl transferase-mediated dUTP-biotin nick end labeling assay

For hematoxylin and eosin (HE) staining, tumor tissues were fixed in 4% formaldehyde, dehydrated with an ethanol gradient, and embedded in paraffin wax. Tissue sections were dewaxed and rehydrated according to a standard protocol, and sections were stained with HE. For the deoxynucleotidyl transferase-mediated dUTP-biotin nick end labeling (TUNEL) assay, apoptotic cells in sections of mouse tumor tissue were detected using an *in situ* apoptosis detection kit (KEYGEN, Nanjing, China) as instructed by the manufacturer. Cells were visualized with a light microscope (Olympus IX70, Tokyo, Japan). The apoptotic index was calculated as follows: apoptotic index = number of apoptotic cells/total number of cells. The *in vivo* experiments strictly obeyed the ethical principles and guidelines for scientific experiments on animals.

### Statistical analysis

Data are expressed as mean ± SE. Statistical significance was determined using *χ*^2^ test, Student’s *t* test, or one-way analysis of variance (ANOVA). Statistical analyses were carried out using SPSS version 13.0 (Chicago, IL, USA) or Origin 7.5 software programs (OriginLab, Northampton, MA, USA). A value of *P* < 0.05 was considered statistically significant.

## Results

### Upregulation of E2F1 is associated with development of MDR in gastric carcinoma

**Figure 1:**
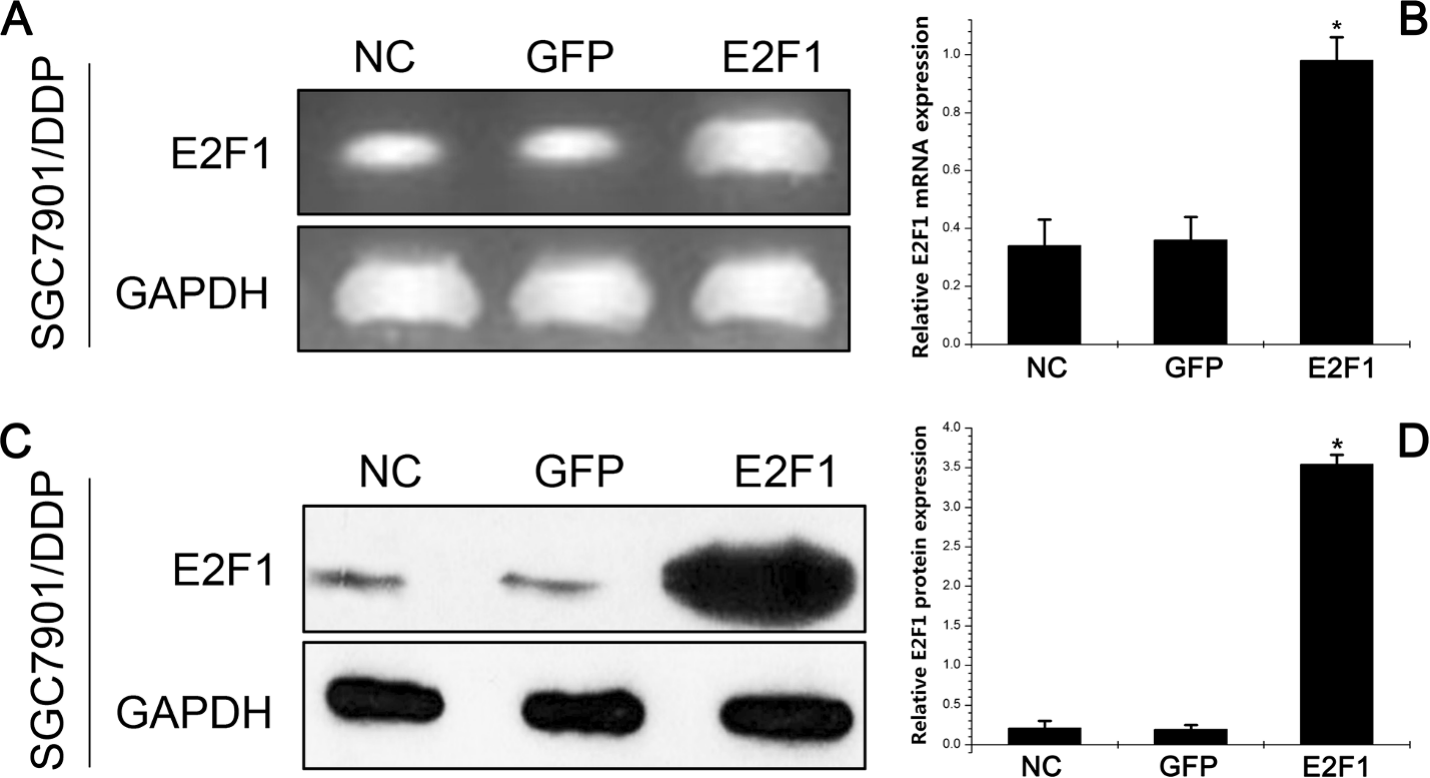
**E2F1 mRNA and protein expressions after gene transfection in SGC7901/DDP cells. A**: Expression level of E2F1 mRNA was determined by semiquantitative reverse-transcriptase polymerase chain reaction; **B**: mRNA results are expressed as the ratio of E2F1 to glyceraldehyde 3-phosphate dehydrogenase (GAPDH); **C**: Expression level of E2F1 protein was determined by western blotting; **D**: Western blotting results are expressed as the ratio of optical density of E2F1 bands to GAPDH bands. All values are mean ± SE. ^*^ *P* < 0.05 for E2F1 group versus GFP group and negative control (NC) group.

To examine the relationship between upregulation of E2F1 and acquisition of MDR in gastric carcinoma, we established gastric carcinoma cells that stably overexpressed E2F1. Transfection of LV-E2F1-GFP into SGC7901/DDP cells led to marked enhancement of E2F1 mRNA (Figure 1A) and protein expression (Figure 1C). Densitometry analysis showed that E2F1 mRNA (Figure 1B) and protein (Figure 1D) levels in the E2F1 group were approximately 3- and 9-fold higher, respectively, than those in the GFP and NC groups (*P* < 0.05). There were no differences in E2F1 levels between GFP and NC groups. These results confirmed that the SGC7901/DDP cells stably transfected with LV-E2F1-GFP showed upregulation of E2F1 mRNA and protein expression.

**Figure 2:**
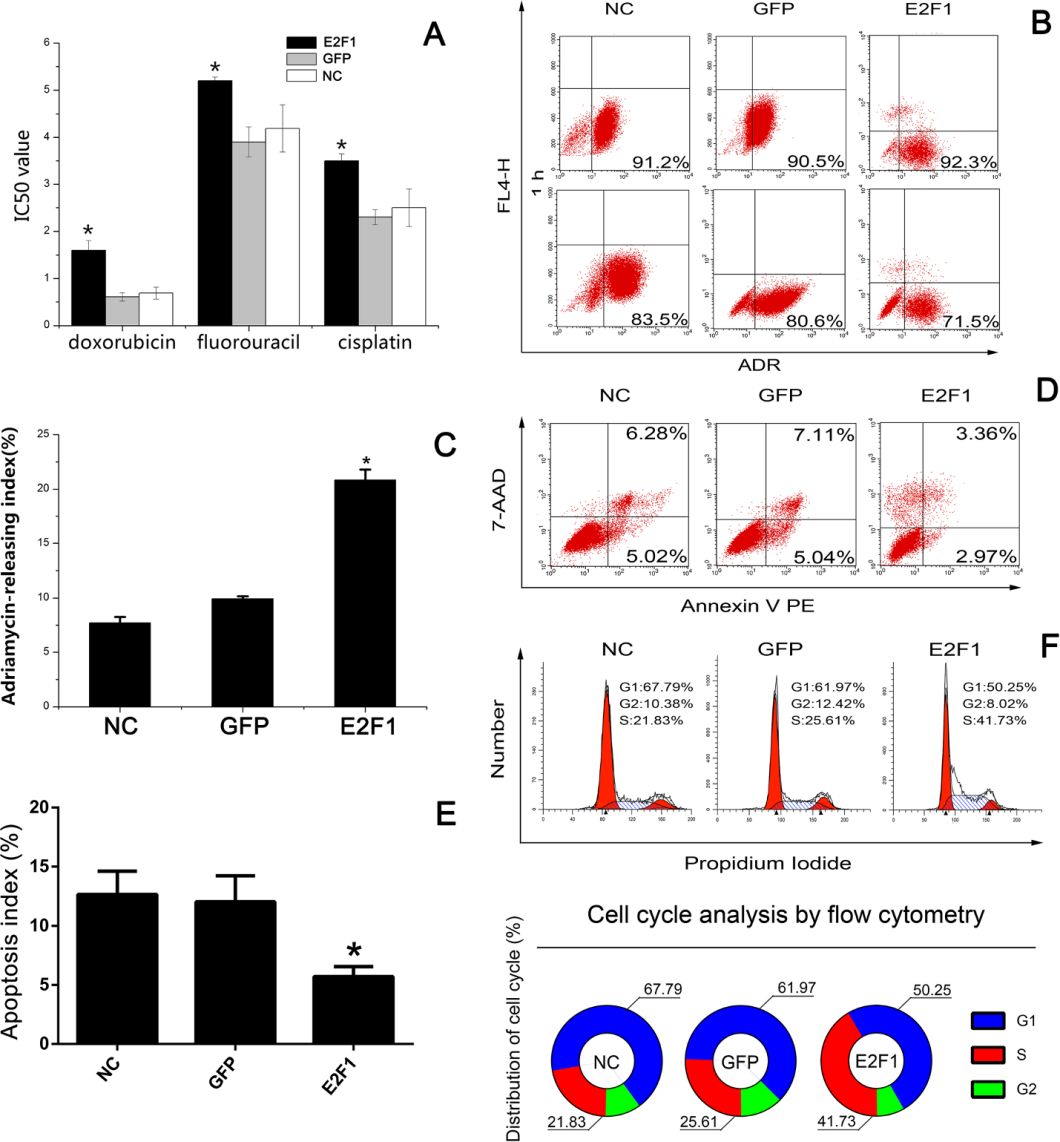
**Effect of upregulation of E2F1 on cell pump rate of doxorubicin, cell cycle, and apoptotic rate in SGC7901/DDP cells. A**: IC50 values for anticancer drugs in SGC7901/DDP cells; **B**, **C**: Pump rate of doxorubicin in SGC7901/DDP cells stably expressing E2F1 was analyzed by flow cytometry; **D**, **E**: Percentages of apoptotic cells were analyzed by flow cytometry. **F**: Cell cycle of SGC7901/DDP cells after E2F1 gene transfection was analyzed by flow cytometry. All values are mean ± SE. ^*^ *P* < 0.05 for E2F1 group versus GFP group and NC group.

We next examined the effects of LV-E2F1-GFP expression on the drug sensitivity of gastric carcinoma cells. Although our SGC7901/DDP cell line was selected under culture with the single anticancer drug cisplatin, these cells also displayed resistance to other anticancer drugs. CCK-8 assay was used to detect the sensitivity of cells to one P-gp-related drug (doxorubicin) and two P-gp-non-related drugs (5-FU and cisplatin). As shown in Figure 2A and Table 1, the cells transfected with LV-E2F1-GFP exhibited significantly increased IC50 values for cisplatin, doxorubicin and 5-fluorouracil compared with the GFP and NC groups (*P* < 0.05). These data indicate that E2F1 upregulation is associated with the MDR phenotype in gastric carcinoma.

**Table 1:** **IC50 values for anticancer drugs in SGC7901/DDP cells**

	Doxorubicin (μg/mL)	5-fluorouracil (μg/mL)	Cisplatin (μg/mL)
E2F1	1.52 ± 0.15^*^	5.22 ± 0.13^*^	3.52 ± 0.15^*^
GFP	0.53 ± 0.09	3.85 ± 1.01	2.26 ± 0.38
NC	0.61 ± 0.12	4.12 ± 0.72	2.52 ± 0.83

IC50 values were evaluated by CCK-8 assay. Each experiment was conducted in triplicate. Data are expressed as means ± SD of four independent experiments. One-way analysis of variance followed by Dunnett’s multiple comparison test revealed statistical differences of *P < 0.05 for the E2F1 group versus the GFP group and NC group.

### Effects of LV-E2F1-GFP on pump rate of doxorubicin

We proposed that upregulation of E2F1 promoted drug efflux in gastric carcinoma *in vitro*. To test this hypothesis, intracellular drug accumulation and retention were evaluated using doxorubicin as a probe that can be detected by flow cytometry. Doxorubicin is a common substrate for P-gp and MRP1, which are involved in well-characterized mechanisms of MDR [11]. As shown in Figure 2B, compared with the GFP and NC groups, the E2F1 group exhibited significantly decreased accumulation and retention of doxorubicin, as well as a higher releasing index of doxorubicin (Figure 2C) (*P* < 0.05).

### LV-E2F1-GFP inhibits apoptosis in the cisplatin-resistant gastric carcinoma SGC7901/DDP cells

Many chemotherapeutic agents exert anticancer activity by inducing apoptosis. Most chemotherapeutic agents applied in the treatment of hematologic malignancies can induce apoptosis, but MDR tumor cells are generally resistant to apoptosis induction [12]. Therefore, we investigated the apoptosis index in cisplatin-resistant gastric carcinoma cells expressing LV-E2F1-GFP. Cells were stained with Annexin V PE and 7-AAD and subsequently analyzed by flow cytometry. The dual parameter fluorescent dot plots present the viable cells in the lower-left quadrant and the apoptotic cells in the right quadrant. Compared with the GFP and NC groups, the E2F1 group exhibited a significantly decreased apoptosis index (5.71% ± 0.86% in E2F1 group compared with 12.04% ± 2.18% and 12.65% ± 1.95% in the GFP and NC groups, respectively; *P* < 0.001). The experiments were repeated three times with three replicates for each group (Figure 2D and E).

We next used flow cytometry to determine whether promotion of MDR by LV-E2F1-GFP in SGC7901/DDP cells was mediated, at least in part, through an effect on cell cycle progression (Figure 2F). We found that the number of cells in S phase in the E2F1 group was markedly increased (41.68% ± 3.24%) compared with the GFP and NC groups (23.74% ± 4.74% and 22.72% ± 3.15%, respectively; *P* < 0.001). Furthermore, the cells in G1 phase were decreased in the E2F1 group (51.33% ± 2.81%) compared with the GFP and NC groups (62.22% ± 3.46% and 65.71% ± 5.00%, respectively; *P* < 0.001). The experiments were repeated three times with three replicates for each group. Together these data indicate that overexpression of E2F1 in SGC7901/DDP cells induced a cell cycle arrest in S phase.

### LV-E2F1-GFP influenced the expression of MDR1, MRP, ZEB1, ZEB2, GAX, and TAp73

**Figure 3:**
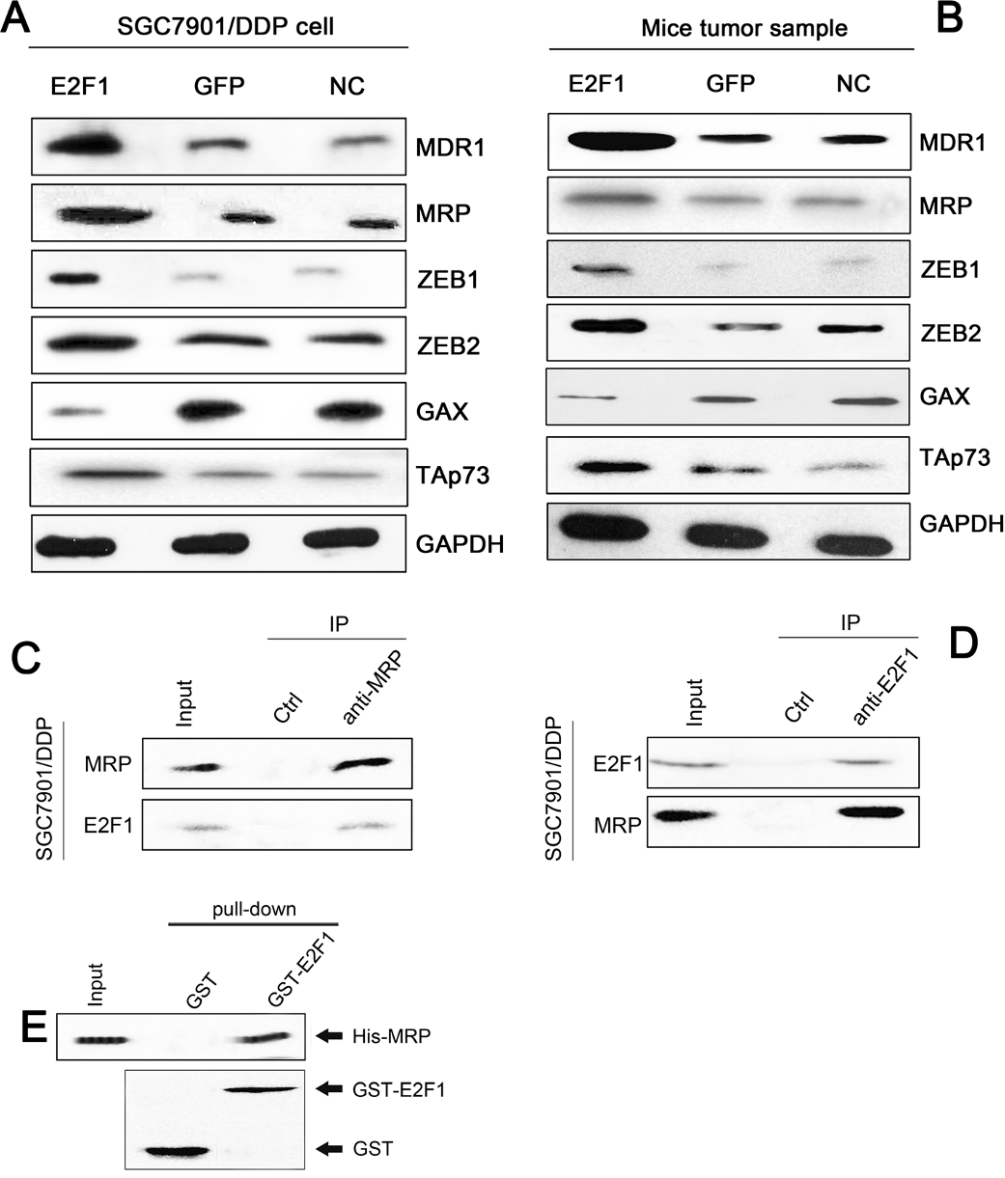
**Overexpression of E2F1 decreased GAX and TAp73, and increased MDR1, MRP, ZEB1, and ZEB2 protein expression. A**, **B**: Protein expression levels of MDR1, MRP, TAp73, GAX, ZEB1, and ZEB2 were determined by western blotting. **C**, **D**: Reciprocal immunoprecipitation assays and western blot analysis of endogenous E2F1 and MRP interaction in SGC7901/DDP cells. **E**: *In vitro* pull-down assays using 6xHis-tagged MRP (His-MRP), GST-E2F1 and GST.

To investigate the mechanism by which LV-E2F1-GFP induces MDR in SGC7901/DDP cells, we evaluated the expression levels of several well-known regulators of apoptosis (Caspase-9, Caspase-3, p53, ZEB1, ZEB2, GAX, and TAp73) and several MDR-related proteins (MDR1, MRP, mTOR and HIF-1α) by western blot. The expression level of GAX protein in the E2F1 group was lower than that in the GFP and NC groups (*P* < 0.05), while the levels of MDR1, MRP, ZEB1, ZEB2 and TAp73 were higher in the E2F1 group than those in the GFP and NC groups (Figure 3A and B).

To better understand the function of E2F1, we performed a yeast two-hybrid screen using E2F1 as the bait. The two-hybrid results identified MRP as an E2F1-interacting protein. To confirm physiological binding, we performed reciprocal immunoprecipitation assays in lysates from the E2F1 stably expressing gastric adenocarcinoma cells and confirmed interaction between endogenous E2F1 and MRP (Figure 3C and D). Furthermore, in pull-down assays using purified proteins, 6xHis-tagged MRP (His-MRP) bound to GST-E2F1, but not GST alone (Figure 3E), confirming MRP as an E2F1-interacting protein and that E2F1 and MRP associate with each other directly.

### Animal studies

**Figure 4:**
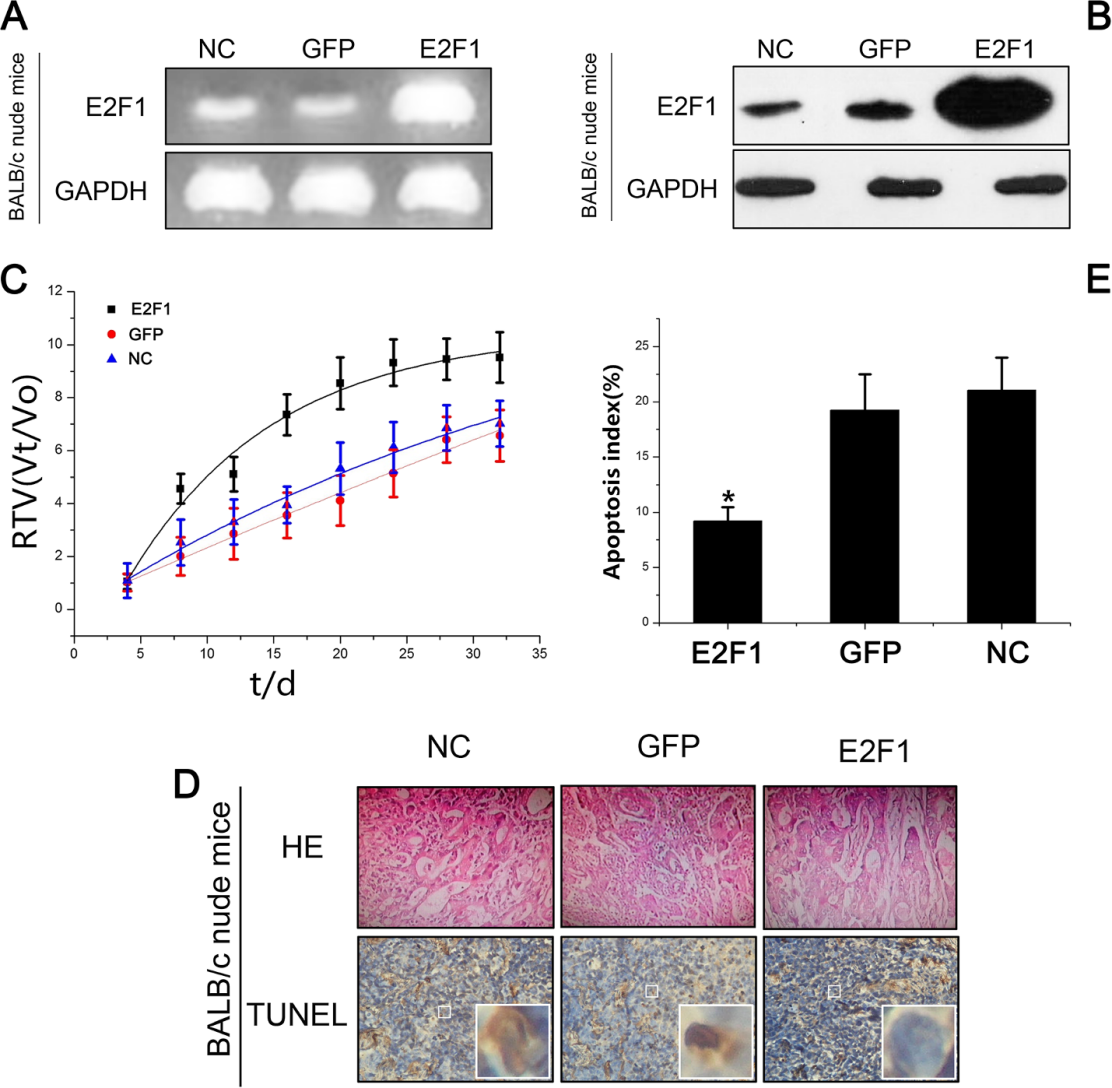
**Apoptosis of LV-E2F1-GFP cells and tumors in nude mice** ***in vivo*** **. A**: mRNA expression level of E2F1 was determined by semiquantitative reverse-transcriptase polymerase chain reaction; **B**: E2F1 protein expression was determined by western blotting; **C**: Relative tumor volume (RTV) of nude mice in each group is presented. Each time point represents the mean RTV for each group. **D**: Tumor cells were evaluated by HE staining and TUNEL assay (× 400); **E**: Percentage of apoptotic cells was analyzed by TUNEL assay. All values are mean ± SE. ^*^ *P* < 0.05 for E2F1 group versus GFP group and NC group.

We next examined the effect of LV-E2F1-GFP on the growth of SGC7901/DDP cells *in vivo*. We implanted SGC7901/DDP cells subcutaneously into the flanks of the BALB/c nude mice to generate SGC7901/DDP tumors. After 7 days, the mice were randomly divided into three groups and administered an intratumoral injection of LV-E2F1-GFP, LV-GFP or PBS as a negative control (NC). Evaluation of expression levels of E2F1 *in vivo* by semiquantitative RT-PCR and western blotting confirmed that the mRNA (Figure 4A) and protein (Figure 4B) expression levels of E2F1 in the E2F1 group were higher than that in the GFP group. Three weeks after implantation, the RTV was significantly higher in the E2F1 group than in the NC and GFP groups (*P* < 0.05) (Figure 4C). As shown in Figure 4D and E, the percentage of apoptotic tumor cells was lower in the E2F1 group at 8.82% ± 1.81%, compared with 19.21% ± 2.3% in the GFP group and 22.13% ± 4.6% in the NC group (*P* < 0.05).

## Discussion

Gastric carcinoma is one of the most common malignancies of humans, with a high incidence in China. While surgical resection remains the primary treatment, chemotherapy is sometimes beneficial in patients with advanced gastric carcinoma. However, the effectiveness of chemotherapy is often thwarted by simultaneous resistance of tumor cells to multiple cytotoxic drugs, known as MDR. MDR currently remains the major obstacle to successful cancer chemotherapy in the clinic [13]. The precise molecular mechanisms underlying MDR remain obscure. However, increasing evidence supports the view that mechanisms involved in MDR include decreased drug accumulation in tumor cells, altered intracellular drug distribution, increased detoxification, diminished drug-target interaction, increased DNA repair, altered cell cycle regulation, and uncoupled pathways linking cellular damage with apoptosis [14, 15].

E2F1 is a member of the E2F family that functions in cell cycle progression and apoptosis induction in response to DNA damage. Recently, we showed that deregulated E2F1 acts as a driving force in gastric carcinoma progression and promotes tumor invasion and metastasis independently from its other cellular activities. Recent evidence, however, showed that high levels of E2F1 and DNp73 downregulate miR-205, which, in turn, controls E2F1 accumulation. Finally, drug resistance associated with this genetic signature is mediated by removing the inhibitory effect of miR-205 on the expression of Bcl-2 and the ATP-binding cassette transporters A2 and A5 related to MDR and malignant progression [16].

One major form of resistance to chemotherapy has been correlated to two molecular pumps, including P-gp, encoded by the MDR1 gene, and MDR protein 1 (MRP1). MDR1 mediates a well-characterized form of drug resistance that is primarily due to overexpression of a P-gp efflux pump [17]. This efflux pump belongs to the ATP-binding cassette (ABC) transporter superfamily and is capable of effluxing many different chemotherapeutic agents, hence the MDR. The resistance is thus due to decreased drug accumulation. E2F1 downregulation has been shown to reverse this form of drug resistance by blocking the efflux pump [9]. Another similar form of MDR due to decreased drug accumulation is MRP1-mediated drug resistance. MRP1 also belongs to the ABC transporter superfamily; however, this efflux pump most likely transports glutathione-conjugated drugs [18]. Our results showed that MDR1 and MRP expression were increased when E2F1 was upregulated. This indicates that E2F1 confers anticancer drug resistance by targeting ABC transporter family members in gastric carcinoma.

In addition to the P-gp and MRP1 signaling pathways, apoptosis also mediates the killing effects of anticancer drugs, which is an important cause of MDR [12]. ZEB1 is a DNA-binding protein that binds to six consensus boxes located within the TAp73 promoter, resulting in the repression of TAp73 transcription [19]. TAp73 is a structural homolog of the p53 tumor suppressor. However, unlike p53, TAp73 is rarely mutated in human tumors and instead is frequently overexpressed. Most studies have shown that TAp73 acts as an apoptosis promoter [20]. Methyl methanesulfonate (MMS) has been shown to induce apoptosis in various cell types through p53/p73-dependent pathways. However, pharmacological and genetic blockade of p53/p73 functions still results in similar or delayed sensitivity to MMS treatment, suggesting the presence of p53/p73-independent apoptotic mechanisms [21]. This may explain the finding that overexpression of E2F1 decreased the percentage of apoptotic cells, thus apoptosis of SGC7901/DDP cells may occur through p53/p73-independent pathways. In addition, growth arrest-specific homeobox (GAX, also known as MEOX2) is a transcription factor originally isolated from vascular smooth muscle. GAX is downregulated by mitogens and upregulated by growth arrest signals, and is also expressed in endothelial cells, where it plays an important role in inhibiting endothelial cell phenotypic changes and the process of angiogenesis [22, 23]. Knowing that ZEB2, a direct target of miR-221 and whose downregulation by miR-221 leads to the upregulation of GAX expression, acts primarily as a transcriptional repressor, Chen *et al*. identified two ZEB2 binding sites in the GAX promoter that modulate the ability of ZEB2 to downregulate GAX promoter activity [24, 25]. Our results showed that the E2F1-overexpression lentiviral vector induced the upregulation of ZEB1, ZEB2, and TAp73 expression and downregulation of GAX. This could explain the decrease of apoptotic cells after E2F1 upregulation in SGC7901/DDP cells.

## Conclusions

In summary, we demonstrated that upregulation of E2F1 significantly inhibited the sensitivity of SGC7901/DDP gastric adenocarcinoma cells to anticancer drugs, and decreased the percentage of apoptotic cells. Upregulation of E2F1 in gastric adenocarcinoma cells potentiated S phase arrest of the cell cycle. Furthermore, our cell line stably expressing E2F1 showed significantly decreased intracellular accumulation of doxorubicin. We conclude that upregulation of E2F1 promotes the development of MDR in gastric carcinoma via inhibition of GAX gene expression, and increased expression of MDR1, MRP, and TAp73. Finally, our observations suggest that E2F1 might serve as a molecular target for the therapy of MDR in gastric carcinoma. We speculate that targeting this gene might aid in the treatment of gastric carcinoma by inhibiting MDR.
